# Advances and challenges in plant N-glycoengineering: when fucosylation matters

**DOI:** 10.3389/fpls.2025.1734060

**Published:** 2025-12-04

**Authors:** Kavya Gauba, Vinny Kunnummel, Alexandra Castilho

**Affiliations:** Institute of Plant Biotechnology and Cell Biology, Department of Biotechnology and Food Science, Universität für Bodenkultur Wien, Vienna, Austria

**Keywords:** plant glycoengineering, fucosylation, N-glycosylation, recombinant glycoproteins, glycan accessibility, plant molecular farming

## Abstract

Plant-based expression systems have emerged as promising platforms to produce recombinant glycoproteins. Transient recombinant protein production is a promising alternative to stable transgenic systems, particularly for emergency situations in which rapid production of novel therapeutics is needed. A potential barrier for plant-based production of therapeutic proteins is that different glycosylation patterns are found on plants. Nevertheless, advances in glycoengineering, particularly in the generation of glycoproteins bearing human- and helminth-like N-glycans, further support the use of plants as valuable systems for biopharmaceutical manufacturing. Glyco-design, including methods to control glycan structures and distributions in plants, is a powerful tool for optimizing the efficacy of therapeutic glycoproteins. However, glycoengineering is not merely a matter of gene knock-in or knock-out and it often requires precise fine-tuning to prevent the formation of aberrant glycan structures. Strategies to address these challenges include: (i) identifying and modulating the activity of proteins/enzymes involved in aberrant glycosylation, (ii) optimizing the subcellular localization and expression levels of glyco-modifying enzymes, (iii) inhibiting glycosidases that trim terminal sugar residues, and (iv) enhancing the accessibility of glycosylation sites to processing enzymes. This review summarizes key developments and challenges in plant N-glycoengineering. Within this broad framework, we highlight core α1,3-fucosylation as a representative case illustrating how a single glycan modification can alter structural accessibility, enzyme activity, and overall glycan maturation.

## 1 Introduction

Protein production in plants can be scaled-up using cell suspension cultures in GMP-compliant bioreactors or whole plants cultivated in greenhouses (Fischer and Buyel, 2020; Huebbers and Buyel, 2021). Plant molecular farming (PMF) has emerged as a powerful platform that harnesses plants as biofactories for recombinant protein production. PMF offers several advantages, including cost-effectiveness, scalability, and inherent biosafety, making it a sustainable and attractive alternative for large-scale protein production. This technology has been successfully applied in the production of industrial enzymes, biopharmaceuticals, and vaccines (Margolin et al., 2020; Shanmugaraj et al., 2020; Goritzer and Strasser, 2021; Eidenberger et al., 2023; Fukuzawa et al., 2024; Yuen et al., 2024). Most therapeutic proteins are glycoproteins. These glycoproteins are decorated with carbohydrate structures (glycans) at asparagine residues within the sequon Asn-X-Ser/Thr, (X≠P)(glycosylation site), through a process called glycosylation. N-glycosylation is a critical post-translational modification that influences protein folding, stability, solubility, and intracellular localization. It is essential for the proper function and pharmacokinetics of glycoproteins and plays a key role in numerous biological processes (Zhou and Qiu, 2019; He et al., 2024).

**Figure 1:**
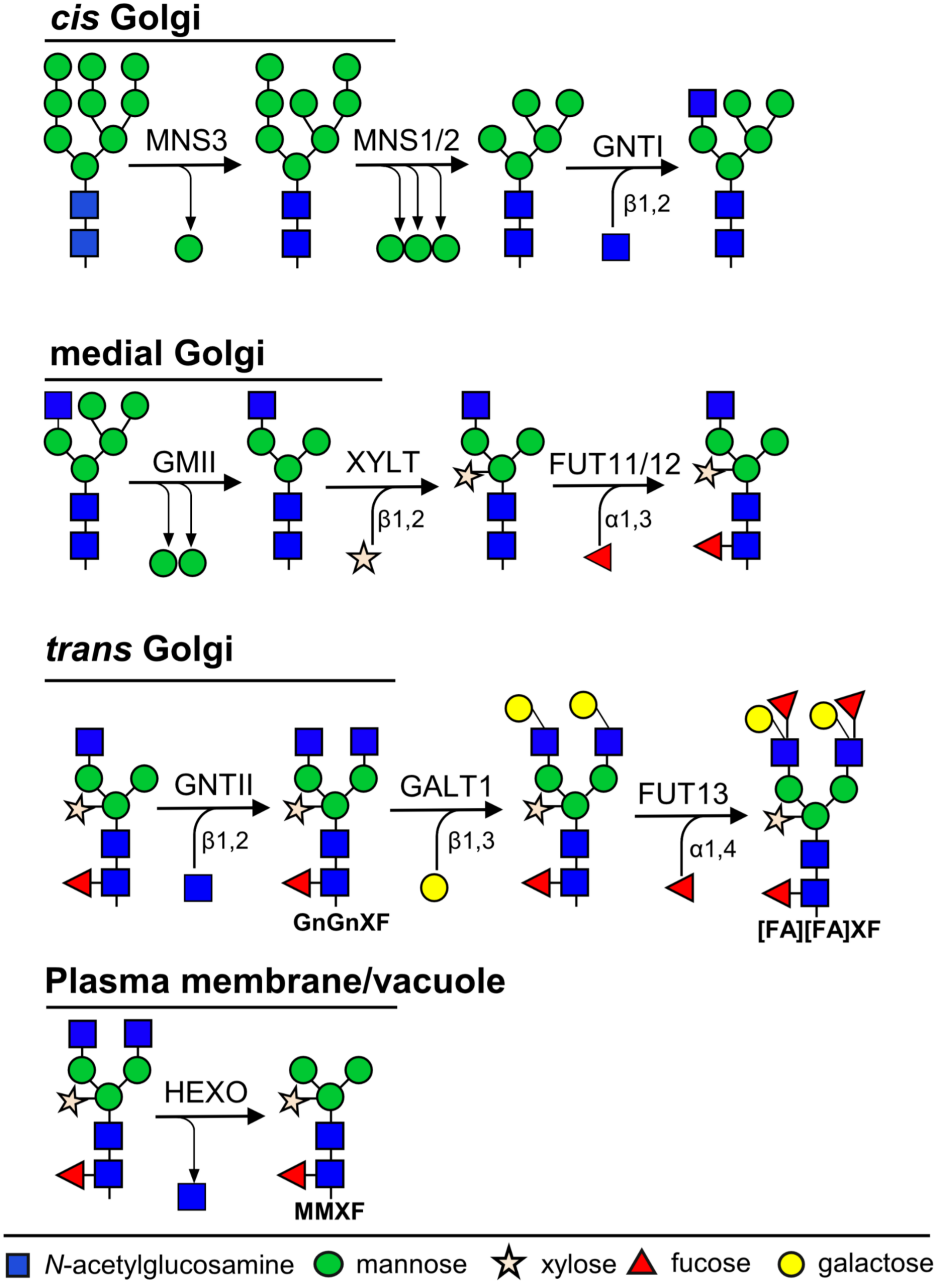
Overview of N-glycosylation pathways in plants. Schematic representation of the sequential steps involved in N-glycan processing within the plant secretory pathway. Following transfer of the oligosaccharide precursor to nascent proteins in the endoplasmic reticulum (ER), glycan maturation proceeds through a series of trimming and extension reactions in the ER and Golgi apparatus. Enzymes are localized to distinct compartments, ensuring the ordered progression from oligomannosidic to complex N-glycans.Abbreviations: MNS3, ER α-mannosidase I; MNS1/2, Golgi α-mannosidase I; GnTI, α1,3-mannosyl-β1,2-N-acetylglucosaminyltransferase I; GMII, Golgi α-mannosidase II; GnTII, α1,6-mannosyl-β1,2-N-acetylglucosaminyltransferase II; XYLT, β1,2-xylosyltransferase; FUT11/12, core α1,3-fucosyltransferases; GALT1, β1,3-galactosyltransferase 1; FUT13, α1,4-fucosyltransferase; HEXO3, β-N-acetylhexosaminidase 3.

Unlike DNA or proteins, glycans are not made from a template but are built step by step by specialized enzymes (glycosyltransferases and glycosidases), transporters, and chaperones expressed in different parts of the cell. Protein glycosylation processes involve sequentially orchestrated modification reactions in the networks of the endoplasmic reticulum (ER) and the Golgi during protein trafficking (Dünser and Schoberer, 2025). In addition, the local cellular environment and the structural accessibility of glycosylation sites further influence the activity and substrate specificity of these enzymes. From the ER to the *cis* Golgi apparatus, the N-glycan processing steps are highly conserved between plants and animals. Significant differences in N-glycan maturation start emerging in the *medial* Golgi and along the secretory pathway, where the activity of plant-specific glycosyltransferases gives rise to complex N-glycans with unique structural features (**Figure 1**). The predominant complex N-glycan in plants is GnGnXF which may be further modified by the addition of Lewis-A epitopes [FA] or by trimming terminal *N*-acetyl-glucosamine (GlcNAc) residues to produce paucimannosidic glycans, (MMXF) (Strasser, 2016) (**Figure 1**, **Supplementary Table S1**). Interestingly, a recent study showed that *N. tabacum* contains two types α1,3-fucosyltransferases, one of which is able to catalyze core fucosylation of high-mannose *N*-glycans generating Man5F glycans (**Supplementary Table S1**) (Navarre et al., 2025).

Compared to mammalian cells, plants have a more limited glycosylation repertoire, often resulting in greater glycan homogeneity but lacking the ability to perform more complex glycan structures. As a result, considerable efforts are being made to overcome the limitations of plant N-glycosylation machinery and to optimize glycosylation pathways for biopharmaceutical production. N-glycoengineering has become a vital strategy to ensure the production of defined and homogeneous glycoforms (Yang et al., 2015; Katoh and Yamamoto, 2021; Nomura et al., 2022), and plants are highly amenable to glycoengineering, enabling the production of recombinant proteins with tailored glycosylation profiles.

The distinct glycan features of plants, such as core α1,3-fucosylation and β1,2-xylosylation, and the absence of certain glycosyltransferases, nucleotide sugar transporters, and interconversion enzymes necessitate customized engineering strategies for therapeutic applications. Notably, transgenic *Nicotiana benthamiana* plants, the most widely used plant species in PMF, have been successfully engineered to (i) eliminate plant-specific β1,2-xylose and core α1,3-fucose residues, (ii) introduce branched GlcNAc residues, and (iii) incorporate terminal β1,4-galactose and α2,6-sialic acid residues (Eidenberger et al., 2023; Kao et al., 2024; Goritzer et al., 2025). Beyond "humanizing" plant glycosylation, there is growing interest in engineering non-human glycoforms such as Helminth-type N-glycans, onto plant-derived glycoproteins with potential therapeutic applications and vaccine development (Wilbers et al., 2017; Bunte et al., 2022; van der Kaaij et al., 2022).

Numerous glycoengineering strategies have been developed, especially for the production of immunoglobulins (IgGs) and derivatives (e.g., Fcabs, scFv-Fc, Fc-fusion proteins) with homogeneous glycosylation profiles (Montero-Morales and Steinkellner, 2018; Castilho and Strasser, 2018; Komarova et al., 2019; Goritzer and Strasser, 2021; Goritzer et al., 2025). However, achieving complete and homogeneous human-like glycosylation in other important glycoproteins, such as hormones, cytokines, receptor extracellular domains, and viral antigens, remains challenging. The success of these efforts largely depends on the number and accessibility of N-glycosylation sites (Loos et al., 2014; Montero-Morales et al., 2019; Castilho et al., 2013; Castilho et al., 2014; Goritzer et al., 2017; Izadi et al., 2025). Here, we highlight advances and persisting challenges in plant N-glycoengineering, redefining the role of plant-specific core α1,3-fucosylation from a perceived obstacle to a strategic engineering handle for optimizing N-glycan conformation, accessibility, and immune-modulating potential.

## 2 Glycoengineering the plant host

Efforts to glycoengineering plant hosts focus on a combination of strategies aimed at suppressing the biosynthesis of plant-specific glyco-epitopes and introducing mammalian glycosyltransferase genes into the plant genome. These approaches enable the glycosylation of recombinant proteins in plant expression systems to more closely resemble that of human cells.

A major achievement in this field has been the downregulation or knockout of genes responsible for the addition of core α1,3-fucose and β1,2-xylose residues. This has led to the establishment of so-called ΔXF plant (cell) lines, which predominantly produce GnGn-type N-glycans on secreted glycoproteins produced in different plant hosts (**Table 1**). Individual knock-out plants for either core α1,3-fucosylation (ΔF) or β1,2-xylosylation (ΔX) genes (Strasser et al., 2008) can also be useful. For example, specific helminths, such as *S. mansoni*, synthesize N-glycans that carry core α1,3-fucose with or without β1,2-xylose (van der Kaaij et al., 2022). Beyond the complete removal of plant-specific glycans, several studies have demonstrated the potential to modulate or reintroduce fucosylation to obtain specific glycan configurations. Heterologous expression of fucosyltransferases from *Zea mays* or *S. mansoni* has been used to alter the linkage specificity and position of core or antennary fucose residues (Castilho et al., 2015; Wilbers et al., 2017; van Noort et al., 2020). These strategies illustrate that glycoengineering can involve not only the elimination of α1,3-fucosylation but also its selective reinstatement to create defined and functionally optimized N-glycan structures.

**Table 1:** Overview of glycoengineered plant host lines used to modulate complex N-glycan processing. The table summarizes commonly used knockout and overexpression lines together with their underlying genetic modifications, characteristic N-glycan profiles, known developmental or physiological phenotypes, and relevant references.

Host	Genetic modification	Resulting N-Glycan profile	Phenotype effects	Key references
ΔXF	Downregulation/Knockout of core α1,3-fucosyltransferase and β1,2-xylosyltransferase	Predominantly GnGn complex N-glycans; widely used as a “humanized” background	Generally, no visible growth defects in several hosts	Strasser et al., 2004; Strasser et al., 2008; Cox et al., 2006; Sourrouille et al., 2008; Li et al., 2016; Mercx et al., 2017; Pedersen et al., 2017; Hanania et al., 2017; Jansing et al., 2019; Jung et al., 2021; Goritzer et al., 2022; Kogelmann et al., 2024
ΔF	Downregulation/Knockout of core α1,3-fucosyltransferase only	Glycans lack α1,3-fucose but retain β1,2-xylose	No visible phenotype in *A. thaliana* and *Nicotiana*;	Strasser et al., 2004; Strasser et al., 2008
ΔX	Downregulation/Knockout of β1,2-xylosyltransferase only	Glycans lack β1,2-xylose but retain core α1,3-fucose	No visible phenotype in *A. thaliana* and *Nicotiana*	Strasser et al., 2004; Strasser et al., 2008
cgl1	Knockout of N-acetylglucosaminyltransferase I (GNTI)	Predominantly Man5 / oligomannosidic N-glycans; absence of complex N-glycans	No visible phenotype in *A. thaliana* and *N. benthamiana*; Developmental defects in rice and Lotus	Vonschaewen et al., 1993; Fanata et al., 2013; Harmoko et al., 2016; Limkul et al., 2016; Pedersen et al., 2017; Herman et al., 2021; Navarre et al., 2025; Strasser et al., 2014
ΔXF^GALT^	Overexpression of β1,4-galactosyltranferase (B4GALT1) targeted to the late Golgi	Predominantly AA N-glycans (β1,4-galactosylation)	Growth defects in *N. benthamiana*	Strasser et al., 2004; Schneider et al., 2015
ΔXF^SIA^	Overexpression of genes necessary for α2,6-sialylation	Predominantly NaNa N-glycans (α2,6-sialylation)	Reduced seed production in *N. benthamiana*	Kallolimath et al., 2016; Kogelmann et al., 2024
ΔXF^GNTIII^	Overexpression of N-acetylglucosaminyltransferase III (GNTIII)	Bisected N-glycans (GnGnbi)	No visible phenotype in *N. tobacco*	Rouwendal et al., 2007
ΔXF^GNTIV^	Overexpression of N-acetylglucosaminyltransferase IV (GNTIV)	Tri-antennary N-glycans ([GnGn]Gn)	No visible phenotype in *N. benthamiana*	Nagels et al., 2011; Nagels et al., 2012
ΔXF^GNTV^	Overexpression of N-acetylglucosaminyltransferase V (GNTV)	Tri-antennary N-glycans (Gn[GnGn])	No visible phenotype in *N. benthamiana*	Nagels et al., 2011; Nagels et al., 2012
ΔXF^GNTIV+V^	Overexpression of GnTIV and V	Tetra-antennary N-glycans ([GnGn][GnGn])	No visible phenotype in *N. benthamiana*	Nagels et al., 2011; Nagels et al., 2012

While plant-specific glycan structures have been associated with potential immunogenicity and allergic reactions, there is currently no clinical evidence of adverse effects in humans (Shaaltiel and Tekoah, 2016; Rup et al., 2017).

An alternative strategy to eliminate plant-specific glycans is the use of hosts lacking GNTI activity, such as the *complex glycan 1* (*cgl1*) mutant (**Table 1**) that predominantly produce oligomannosidic N-glycans (Man5, **Supplementary Table S1**). Arabidopsis *cgl1* mutants show no visible growth or developmental phenotypes (Vonschaewen et al., 1993; Strasser, 2014), in contrast to *Oryza sativa* (rice) and *Lotus japonicus* GNTI mutants, which exhibit severe developmental and reproductive defects (Fanata et al., 2013; Harmoko et al., 2016).

*N. benthamiana* tolerates the removal of plant-specific complex N-glycans well; no obvious phenotypic abnormalities have been reported in lines lacking xylosyltransferase and fucosyltransferase activity (Strasser et al., 2008; Kogelmann et al., 2024). Furthermore, the stable expression of mammalian glycosyltransferases such as N-acetylglucosaminyltransferases III, IV, and V to produce glycoproteins with bisected and branched N-glycans does not significantly impact plant growth or morphology (Rouwendal et al., 2007; Nagels et al., 2011) (**Table 1**). However, developmental phenotypes have been observed in transgenic *N. benthamiana* plants expressing α1,3/4-Lewis fucosyltransferase (Joly et al., 2004), β1,4-galactosyltransferase (ΔXF^GALT^) (Schneider et al., 2015; Kogelmann et al., 2024), and α2,6-sialyltransferase (ΔXF^SIA^) (Kallolimath et al., 2016). Notably, plants expressing human β1,4-galactosyltransferase and genes required for sialylation display pronounced growth defects and a marked reduction in seed production (Schneider et al., 2015; Kallolimath et al., 2016; Kogelmann et al., 2024) (**Table 1**). To overcome this shortcoming a recent study has established plant cell packs (PCPs) derived from ΔXF^SIA^ transgenic plants that enable transient expression of recombinant proteins carrying sialylated N-glycans (Dianatkhah et al., 2025).

Overall, beyond their applications in biopharmaceutical production, glycoengineered plant hosts provide valuable tools for elucidating the functional roles of protein glycosylation in plant growth, development, and stress responses (Kang et al., 2008; Liu et al., 2018; Strasser, 2014; Strasser et al, 2021; Strasser, 2022).

## 3 Transient glycoengineering

Despite advances in generating transgenic plant lines with engineered glycosylation pathways, PMF still predominantly relies on transient expression systems to modulate N-glycosylation of recombinant proteins.

*Rhizobium radiobacter* (formerly *Agrobacterium tumefaciens*) is used as a gene delivery vector by agroinfiltration, enabling high-efficient protein expression without stable genetic transformation (Chincinska, 2021). The remarkable plasticity of the plant glycosylation machinery allows for the *de novo* design and synthesis of novel glycans, difficult to achieve via chemoenzymatic methods or alternative expression systems.

**Figure 2:**
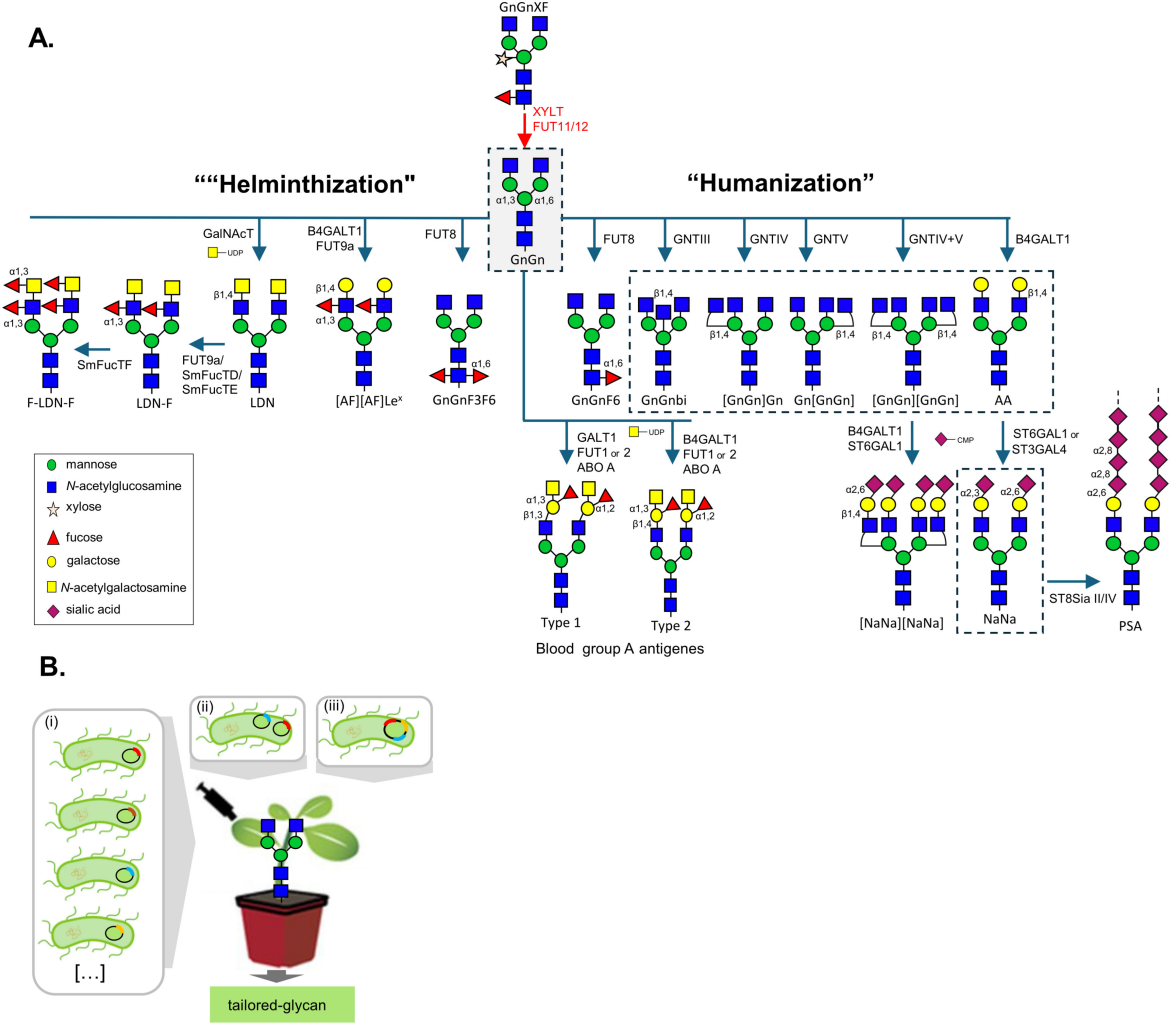
Strategies for generating helminth- and human-like N-glycans in plants. **(A)** Targeted knockout of *XYLT* and *FUT* (red arrows) produces the ΔXF plant line, which synthesizes complex N-glycans of the GnGn type. This “glycan template” provides a foundation for further modification through the transient single or multiple expression of heterologous mammalian or helminth glycosyltransferases (blue arrows). Such engineered enzymes introduce branching, bisecting, fucosylation, galactosylated or sialylation patterns, enabling the production of highly customized glycoforms. Glycan structures enclosed in dashed boxes represent glycan structures that have also been obtained in stably transformed plant lines. N-glycan structures were labelled according to the ProGlycAn nomenclature (Proglycan_nomenclature_2023.pdf) **(B)** Multiple transient expression (MUTE) of glycosylation enzymes in plants can be implemented by: (i) co-infiltrating several *Agrobacterium* strains, each carrying a single expression cassette; (ii) assembling multiple expression constructs within one *Agrobacterium* strain; or (iii) introducing a single vector containing multiple gene expression cassettes. Abbreviations: GNTIII, β1,4-mannosyl-β1,4-N-acetylglucosaminyltransferase III; GNTIV, α1,3-mannosyl-β1,4-N-acetylglucosaminyltransferase IVa; GNTV, α1,6-mannosyl-β1,6-N-acetylglucosaminyltransferase V; FUT8, α1,6-fucosyltransferase; FUT1/2, α1,2-fucosyltransferases; FUT9a, α1,3-fucosyltransferase IXa; B4GALT1, β1,4-galactosyltransferase; ST6GAL1, α2,6-sialyltransferase; ST3GAL4, α2,3-sialyltransferase; ABO-A, α1,3-N-acetylgalactosaminyltransferase; GalNAcT, α1,4-N-acetylgalactosaminyltransferase; ST8Sia II/IV, α2,8-sialyltransferases II and IV; SmFucT D/E/F, *Schistosoma mansoni* α1,3-fucosyltransferases D, E, and F.

A particularly innovative approach is the multiple transient expression system (MUTE) in *N. benthamiana*, where agrobacteria carrying different expression constructs are co-infiltrated to deliver reporter glycoproteins along with glycosyltransferases, nucleotide sugar interconversion enzymes, and transporters required to reprogram the plant glycosylation pathway (Loos and Castilho, 2015). This modular system enables the *in-planta* assembly of synthetic glycosylation pathways in a controlled and flexible manner. MUTE has been successfully applied to produce recombinant proteins with tailored glycosylation (**Figure 2A**). Engineering certain glycoforms can be relatively straightforward, requiring only the overexpression of enzymes that are absent or "missing" in plants. For example, overexpression of the core α1,6-fucosyltransferase (FUT8); β1,4-galactosyltransferase (B4GALT1); β1,4-*N*-acetyl-glucosaminyltransferase III (GNTIII); α1,3-mannosyl-β1,4-N-acetylglucosaminyltransferase (GNTIV) or α1,6-mannosyl-β1,6-N-acetylglucosaminyltransferase (GNTV) has enabled the synthesis of human-like core-fucosylated, galactosylated, bisected and tri-antennary N-glycans, respectively (**Figure 2A**). In contrast, other modifications require configuration of the plant N-glycosylation machinery to reconstruct entire biosynthetic pathways, such as those needed for human-type protein sialylation (Castilho et al., 2010; Castilho et al., 2012; Bohlender et al., 2020; Izadi et al., 2023a); for the synthesis of helminth-like mono- and bi-fucosylated β14GalNAc-GlcNAc (LacdiNAc or LDN) motifs (LDN-F/F-LDN-F, **Figure 2A**, **Supplementary Table S1**) (Wilbers et al., 2017; Bunte et al., 2022; van Noort et al., 2020; van der Kaaij et al., 2022) and for blood group antigens (Beihammer et al., 2021). One striking example is the coordinated transient co-expression of eleven mammalian proteins involved in glycan branching, galactosylation, and sialylation in an *N. benthamiana* ΔXF background, which enabled the production of recombinant human erythropoietin (EPO) carrying bi-, tri-, and tetra-sialylated complex N-glycans as well as sialylated mucin-type O-glycans, devoid of plant-specific residues (Castilho et al., 2012; Castilho et al., 2013). Despite the remarkable achievements, multiple transient expression methodology can be prone to batch-to-batch inconsistencies. Therefore, binary expression vectors tailored to accommodate multiple genes encoding glycosyltransferases and/or glycosylhydrolases, along with various genetic elements are being optimized, hereby enhancing the flexibility to manipulate N-glycan structures in plant transient expression systems (Castilho et al., 2013; Schneider et al., 2014; Bohlender et al., 2020; Kittur et al., 2020; Mukherjee et al., 2021; Izadi et al., 2023b; Kao et al., 2024) (**Figure 2B**).

Finally, studies evaluating the impact of glycosylation on protein function traditionally relied on site-directed mutagenesis to remove N-glycosylation sites, thereby preventing glycan attachment. However, such mutations can alter protein folding, conformation, or stability, making it difficult to distinguish the direct effects of glycosylation from those arising due to structural perturbations. As an alternative approach, the synthesis of mannosidic glycans can be induced by treatment with kifunensine (KIF), a potent α-mannosidase I inhibitor, which blocks N-glycan processing at the Man_9_GlcNAc_2_ stage. When combined with the overexpression of an Endo H enzyme targeted to the late Golgi, this strategy enables the production of deglycoproteins, without the need for mutating the protein sequence (Izadi et al., 2023a; Izadi et al., 2025. This method provides a powerful tool to investigate the functional consequences of glycosylation while preserving native protein structure and folding.

## 4 Fine-tuning the N-glycan processing

Glycosylation is tightly regulated across multiple levels, including gene expression, protein folding, intracellular trafficking, and the activity and localization of glycosyltransferases and glycosidases. Engineering recombinant protein glycosylation is often complicated by the number and accessibility of glycosylation sites. “Humanization” and "helminthization" of N-glycans in plants by glycoengineering seems to be hampered by the formation of aberrant glycan structures and often requires fine-tuning to (i) optimize the expression and subcellular localization of glyco-modifying enzymes (Strasser et al., 2009; Castilho et al., 2011b; Wilbers et al., 2017; Kallolimath et al., 2018; Konig-Beihammer et al., 2021), (ii) inhibit glycosidases that trim terminal sugar residues (Shin et al., 2017; Kriechbaum et al., 2020; Blumberg et al., 2025) (iii) modulate enzyme activity, and (iv) improve the glycosite accessibility to processing enzymes (Castilho et al., 2015; Wilbers et al., 2017).

**Figure 3:**
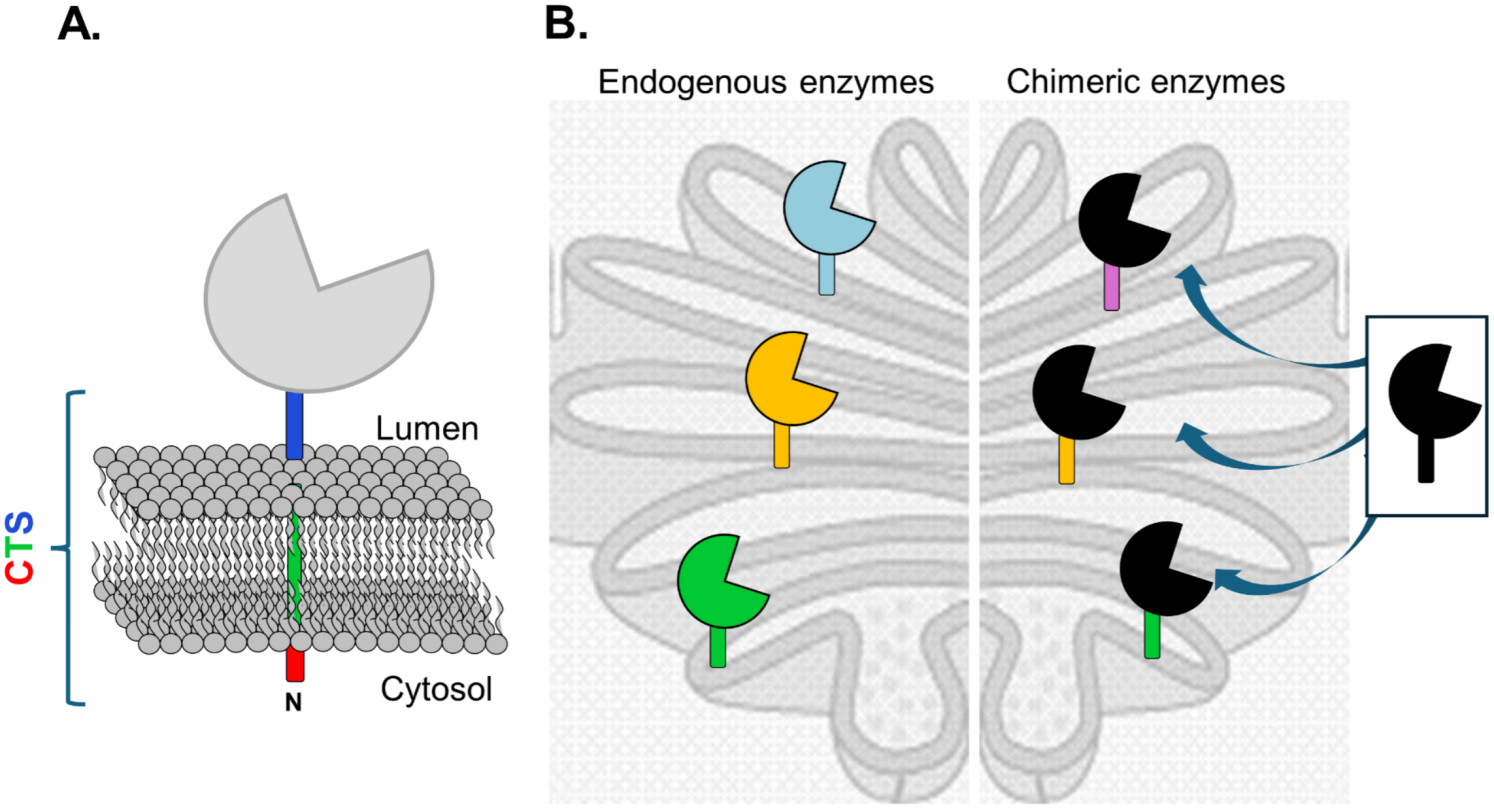
Domain swapping of glycosyltransferases (GTs) to control sub-Golgi localization. **(A)** Glycosyltransferases (GTs) are typically type II membrane proteins composed of an N-terminal cytosolic tail, a transmembrane domain, and a luminal stem region (collectively referred to as the CTS domain). The CTS region is a key determinant of the enzyme’s sub-Golgi localization, directing it to the cis-, medial-, or trans-Golgi compartments. **(B)** Swapping CTS regions between different GTs enables the rational reorganization of the glycosylation pathway. The resulting chimeric GT localizes to the Golgi subcompartment defined by the CTS region of the donor enzyme, thereby allowing fine-tuned control over glycan maturation.

N-glycan processing enzymes are arranged along the plant secretory pathway, where type II membrane-bound glycosyltransferases/glycosidases act in a stepwise manner (Schoberer and Strasser, 2011). These enzymes contain a CTS (cytoplasmic-transmembrane-stem) domain, that governs their sub-cellular localization, and an enzymatic domain (Sasai et al., 2001; Tu and Banfield, 2010; Welch and Munro, 2019) (**Figure 3A**). While the exact size of the stem region of the CTS domain can vary, it generally spans a significant portion of the protein between the transmembrane domain and the catalytic domain. Besides regulating the localization, the stem region is also required for multimer formation and important for homophilic interactions with other glycosyltransferases (Welch and Munro, 2019; Tomida et al., 2022). Localization within Golgi cisternae (cis, medial, trans/TGN) reflects the enzyme sequential role in glycan maturation, although overlap between sub-compartments is common (Schoberer and Strasser, 2011). Recent insights identified molecular codes within CTS regions as key determinants of this localization (Yagi et al., 2024).

In plants, a clear separation of early- (MNS1, GNTI, GMII, GNTII, XYLT, and FUT11/12) from late-acting enzymes (GALT1, FUT13) has been established but the existence of a distinct medial-Golgi or intermediate location is questionable (Schoberer and Strasser, 2011).

Despite conserved localization signals across species, subtle differences in sub-Golgi targeting have been observed for heterologous glycosyltransferases expressed in plants (Saint-Jore-Dupas et al., 2006). Miss localization of heterologous enzymes can disrupt native pathways, leading to hybrid or incomplete glycan structures. For instance, overexpressing full-length GNTIII, B4GALT1, or α1,3-fucosyltransferase IXa (FUT9a) disrupted the activity of endogenous GNTII, GMII and XYLT (Bakker et al., 2001; Rouwendal et al., 2007, Rouwendal et al., 2009; Wilbers et al., 2017; Nguyen et al., 2023). To overcome this, domain-swapped chimeric enzymes with Golgi-targeting CTS domains have been engineered (**Figure 3B**, **Table 2**) (Loos and Steinkellner, 2014; Strasser et al., 2014), enabling precise sub-Golgi localization and control over glycosylation (Strasser et al., 2009; Castilho et al., 2011a; Castilho et al., 2011b; Dicker et al., 2015; Wilbers et al., 2017; Bohlender et al., 2020; van Noort et al., 2020).

**Table 2:** Representative type II glycosyltransferases and glycosidases whose cytoplasmic, transmembrane, and stem (CTS) domains serve as targeting signals for specific subcellular localization of heterologous enzymes. UniProt accession numbers and amino acid ranges defining the CTS or CT domains are indicated.

Subcellular targeting	Type II transmembrane protein	Acc. UniProtKB	CTS length (aa)
ER	*Arabidopsis thaliana* α-glucosidase I (GCSI)	F4HTM3	1-90
ER/cis-Golgi	*Arabidopsis thaliana* Golgi α1,2-mannosidase A (MNS1A)	Q9C512	1-88
	*Arabidopsis thaliana N-a*cetylglucosaminyltransferase I (GNTI)	Q9XGM8	1-77
cis/medial-Golgi	*Arabidopsis thaliana* Golgi α1,2-mannosidase C (MNS1C)	Q93Y37	1-109
	*Arabidopsis thaliana* Golgi mannosidase II (GMII)	Q9LFR0	1-92
	*Arabidopsis thaliana* β1,2 xylosyltransferase (XYLT)	Q9LDH0	1-90
medial-Golgi	*Arabidopsis thaliana* α1,3-fucosyltransferase (FUT11)	Q9LJK1	1-66
	*A. thaliana* β1,2-galactosyltransferase (MUR3)	Q7XJ98	1-120
	*Schistosoma mansoni* α1,3-fucosyltransferase (FucTC)	E2EAI6	1-35 (CT)
Medial/trans-Golgi	*Arabidopsis thaliana* β1,2-*N-*acetylglucosaminyltransferase II (GNTII)	Q9FT88	1-76
	*Homo sapiens* β1,4-galactosyltransferase (B4GALT1)	P15291	1-68
	*Arabidopsis thaliana* Xyloglucan β1,2 galactosyltransferase (MUR3)	Q7XJ98	1-120
	*Homo sapiens* α1,6-fucosyltransferase (FUT8)	Q9BYC5	1-108
trans-Golgi	*Homo sapiens* α1,4-fucosyltransferase (FUT9)	Q9Y231	1-60
	*Arabidopsis thaliana* α1,3-galactosyltransferase (GALT1)	Q8L7F9	1-60
	*Arabidopsis thaliana* α1,4-fucosyltransferase (FUT13)	Q9C8W3	1-52
	*Physcomitrium patens* α1,4-fucosyltransferase (FT4)	A9T1L4	1-130
	*Rattus norvegicus* α2,6-sialyltransferase (ST6GAL1)	P13721	1-52
	*Homo sapiens* α2,3-sialyltransferase (ST3GAL4)	Q11206	1-116
	*Homo sapiens* α2,8 sialyltransferase (ST8SIA2)	Q92186	1-50
Plasma membrane	*Arabidopsis thaliana* β-hexosaminidase 3 (HEXO 3)	Q8L7S6	1-26 (CT)

Multiple examples of CTS swapping are available in the literature (**Table 3**) (McGinness et al., 2025). An important one was the replacement of the endogenous CTS of the human B4GALT1 by the rat α2,6-sialyltransferase (ST6GAL1) or GALT1 instead of the medial-Golgi targeting CTS domains previously used (Bakker et al., 2001, Bakker et al., 2006; Rouwendal et al., 2009) to avoid synthesis of hybrid structures. Targeting B4GALT1 to the late Golgi compartment (^ST6^B4GALT1, ^GALT1^B4GALT1 and ^FT4^B4GALT1) is required for the synthesis of biantennary galactosylated (AA)_;_ sialylated (NaNa); Lewis-X (Le^X^, [AF]) and blood type antigen epitopes (**Figure 2A**, **Supplementary Table S1**) (Castilho et al., 2010; Strasser et al., 2009; Wilbers et al., 2017; Goritzer et al., 2019; Kallolimath et al., 2020; Kriechbaum et al., 2020; Bohlender et al., 2020; Beihammer et al., 2021). Also, accurate mimicry of *Schistosoma mansoni* N-glycosylation in plants requires a strict sequential order of core fucosylation, wherein α1,6-fucosylation must precede α1,3-fucosylation to prevent interference with the activity of the introduced fucosyltransferases (FucTs) (van Noort et al., 2020). While this strategy has been effective, species-specific differences must be considered (Hesselink et al., 2014; Dicker et al., 2015).

**Table 3:** Chimeric glycosyltransferases with swapped cytoplasmic–transmembrane–stem (CTS) regions used to achieve specific target glycoforms, either individually or in co-expression experiments. (“^CTS^GT”).

Chimeric proteins	Target glycan	Ref
^GMI^mRFP; ^GNTI^mRFP	Man5	Schoberer et al., 2023
^FUT11^GNTIV; ^XylT^GNTIV	[GnGn]Gn	Nagels et al., 2011; Castilho et al., 2011b
^FUT11^GNTV; ^XylT^GNTV	Gn[GnGn]	Nagels et al., 2011; Castilho et al., 2011b
^GMII^GNTIII; ^XylT^GnTIII; ^FUT11^GNTIII; ^ST6^GNTIII	GnGnbi	Castilho et al., 2011b; Rouwendal et al., 2007
^FUT11^GNTIV+ ^FUT11^GNTV+ ^ST6^GNTIII	[GnGn][GnGn]bi	Castilho et al., 2011b
^FUT11^FUT8	GnGnF(6)	Castilho et al., 2011a; Forthal et al., 2010; Wilbers et al., 2016
^ST6^B4GalT1+ ^ST6^FUT9a	Le^x^	Wilbers et al., 2017
^FT4^B4GALT1; ^GALT1^B4GALT1; ^ST6^B4GALT1	AA	Strasser et al., 2009; Kriechbaum et al., 2020; Bohlender et al., 2020
^ST6^FUT1/2+ ^ST6^B4GALT1+^ST6^ABO-A	ABH(0) antigens	Beihammer et al., 2021
^ST6^B4GALT1+ST6GAL1; ^FT4^B4GALT1+^FT4^ST6GAL1	NaNa^α2,6^	Castilho et al., 2010; Bohlender et al., 2020
^ST6^B4GALT1+ST3GAL4	NaNa^α2,3^	Kallolimath et al., 2016
^FUT11^GNTIV+ ^FUT11^GNTV+ ^ST6^B4GALT1+ST6GAL1	[NaNa][NaNa]	Castilho et al., 2013

Besides swapping CTS regions, the expression level of the heterologous glycosyltransferase is also an important factor and often fine-tuning the enzyme expression is required to increase the homogeneity of glycosylation on recombinant glycoproteins (Dicker et al., 2015; Wilbers et al., 2017; Kallolimath et al., 2018). It has become clear that even with the appropriate CTS region, expression levels over a certain threshold can miss target the enzyme and negatively impact the generation of fully processed N-glycans. Indeed, studies conducted with B4GALT1 targeted to the required late Golgi compartment (^ST6^B4GALT1) still showed the importance of choosing the right promoter to optimize expression and avoid aberrant glycosylation (Wilbers et al., 2017; Kallolimath et al., 2018; Bohlender et al., 2020).

Another aspect that needs attention when transiently expressing recombinant proteins is the possible “overloading” of the secretory pathway which can interfere with the activity of endogenous Golgi-resident enzymes leading to incomplete glycosylation (truncated and hybrid glycans). This is well illustrated when MUTE is used to produce multi-sialylated (Castilho et al., 2014; Schneider et al., 2014; Izadi et al., 2023a) or multi-fucosylated recombinant proteins in plants (van Noort et al., 2020). Some recombinant proteins expressed in plants are decorated with mono-antennary N-glycans (MGn, MA, MNa, single Le^x^ and LDN-F motifs, **Figure 2A**, **Supplementary Table S1**). The presence of N-glycan structures with a single terminal GlcNAc residue has been attributed to a partial inhibition of endogenous GNTII since co-expression of recombinant GNTII results in a significant increment of fully processed glycans (Loos et al., 2014; Schneider et al., 2014; Dicker et al., 2016; Goritzer et al., 2017).

Substantial progress has been achieved in engineering plant glycosylation machinery; however, the efficacy of these approaches remains protein-specific, as outcomes are strongly influenced by the local structural context and accessibility of individual N-glycosylation sites due to protein conformation. Exposed glycosylation sites are more accessible to glycan-modifying enzymes (glycosyltransferases and glycosidases), leading to greater glycan heterogeneity. In contrast, buried glycosylation sites are more protected from glycosidases but also less accessible to glycosyltransferases, often resulting in incomplete glycan processing. For example, full antibodies, particularly IgGs, have a conserved N-glycosylation site at Asn297 buried within the CH2 domain of Fc region. Although there is no direct information comparing the exposure of the glycosylation site Fcabs (Fc fragment with engineered antigen binding sites) and in full antibodies, some relevant insights can be inferred by comparing glycoengineering of Fcabs and full antibodies (Kriechbaum et al., 2020). Site-specific N-glycan processing has been observed for several glycoproteins with multiple glycosylation sites (GS). Good examples are IgM and IgE antibodies with 5 and 7 GS, respectively. While most GS carry complex GnGn/MGn glycans (IgM : GS1–3 and IgE: GS1-5), those at the CH3 domain are decorated with oligomannosidic structures (Loos et al., 2014; Montero-Morales et al., 2019; Jugler et al., 2022). The structural polypeptide features that affect these modifications are not yet understood.

### 4.1 Paucimannosidic N-glycans

Proteins trafficking through the Golgi apparatus are subjected to processing by various glycosyltransferases. Most recombinant proteins expressed in plants acquire complex N-glycans, with GnGnXF or GnGn as the predominant forms, depending on whether the host is wild-type or glycoengineered ΔXF plants, respectively. However, the removal of terminal GlcNAc residues can lead to truncated N-glycans such as MMXF or MM, collectively referred to as paucimannosidic glycans (**Figure 1**, **Supplementary Table S1**).

The formation of these structures is primarily mediated by β-*N*-acetylhexosaminidases particularly HEXO1 and HEXO3 (Strasser et al., 2007a; Liebminger et al., 2011). Among them, HEXO3, localized at the plasma membrane, has been identified as the main enzyme responsible for trimming terminal GlcNAc and N-acetylgalactosamine (GalNAc) (Strasser et al., 2007b; Liebminger et al., 2011; Shin et al., 2017; Alvisi et al., 2021). In contrast, HEXO2 exhibits strict β-galactosaminidase activity (Alvisi et al., 2021).

Recombinant proteins expressed in *N. benthamiana* exhibit varying sensitivity to β-hexosaminidase activity. For instance, in full IgG1 antibodies and Fc fragments, the N-glycosylation site buried within the CH2 domain is largely protected from enzymatic trimming. Conversely, glycoproteins such as bovine follicle-stimulating hormone (Dirnberger et al., 2001), human α1-antitrypsin (A1AT) (Castilho et al., 2014), human secreted alkaline phosphatase (SEAP) (Becerra-Arteaga and Shuler, 2007), and the helminth glycoprotein Omega-1 (Wilbers et al., 2017) have shown a high proportion of paucimannosidic glycans.

These truncated glycans are often considered undesirable in therapeutic applications due to their potential to increase immunogenicity and adverse immune responses. Consequently, several strategies have been developed to suppress their formation. These include (i) the use of *Arabidopsis thaliana hexo3* knockout mutants (Castilho et al., 2014), (ii) RNA interference to silence HEXO3 (Shin et al., 2017) and (iii) CRISPR-based genome editing of β-hexosaminidase genes (Blumberg et al., 2025).

An alternative strategy involves capping terminal GlcNAc and GalNAc residues with fucose using FUT9a. This strategy was used to make glycans resistant to hexosaminidase activity and enhanced the accumulation of fucosylated LDN motifs in helminth glycoproteins (Wilbers et al., 2017). Glycan processing by endogenous glycosidases should in most cases be avoided, but sometimes the action of glycosidases can be beneficial to fine-tune a specific glycan structure. Interestingly, paucimannosidic glycans can also be advantageous. Exposed mannose residues promote rapid receptor-mediated clearance from the bloodstream (Yang et al., 2015) and have shown enhanced efficacy in mannose receptor (MR)-mediated cellular uptake of biopharmaceuticals (Limkul et al., 2016; Sariyatun et al., 2021). Thus, for certain therapeutic proteins, especially those used in enzyme replacement therapy (ERT), paucimannosidic N-glycans are desirable. To enrich these glycans in plant-derived ERT proteins (e.g., human β-glucocerebrosidase, acid α-glucosidase), proteins have been targeted to the plant storage vacuole, where terminal residues are naturally removed to produce paucimannosidic glycans (Shaaltiel et al., 2007; Liebminger et al., 2011; Tekoah et al., 2013). Additionally, glycoengineered *A. thaliana* mutants lacking ALG3 (an α1,3-mannosyltransferase involved in early N-glycan precursor assembly) have been used to produce recombinant human acid α-glucosidase with predominantly MM-type glycans (Sariyatun et al., 2021).

A landmark in the field is taliglucerase alfa, a β-glucocerebrosidase produced in carrot cell suspension cultures by Protalix Biotherapeutics (Carmiel, Israel). ELELYSO® was the first plant-made pharmaceutical approved by the U.S. Food and Drug Administration (FDA) for human use.

As noted, sensitivity to β-hexosaminidase trimming is highly correlated with the accessibility of glycosylation sites. While glycosylation sites on proteins such as A1AT are fully accessible to β-hexosaminidase activity, others like the Asn297 buried in the CH2 domain of antibodies are structurally shielded from enzymatic access. Interestingly, the presence of core α1,3-fucose appears to enhance the exposure of certain glycans to β-hexosaminidases. Co-expression of FUT11 significantly increases the formation of paucimannosidic glycans in Fcabs (MMF, **Supplementary Table S1**), but not in full antibodies. This observation suggests that structural rearrangements in Fcabs may expose the glycosylation site, rendering it more susceptible to glycan trimming, particularly when core fucosylation alters glycan conformation and enhances accessibility to glycosidases. Of note, enhancing the synthesis of paucimannosidic N-glycans by core fucosylation is not observed when the α1,6-fucosyltransferase is used (Shin et al., 2017).

### 4.2 Man5 and hybrid N-glycans

Man5 and hybrid N-glycans play crucial roles in both normal physiology and disease. Man5 structures are central to glycoprotein quality control, structural integrity, and therapeutic relevance, while hybrid N-glycans contribute to neuronal function, immune modulation, and protein stability. Alterations in either glycoform are linked to pathological processes, underscoring their biological and biomedical importance (Hall et al., 2016).

**Figure 4:**
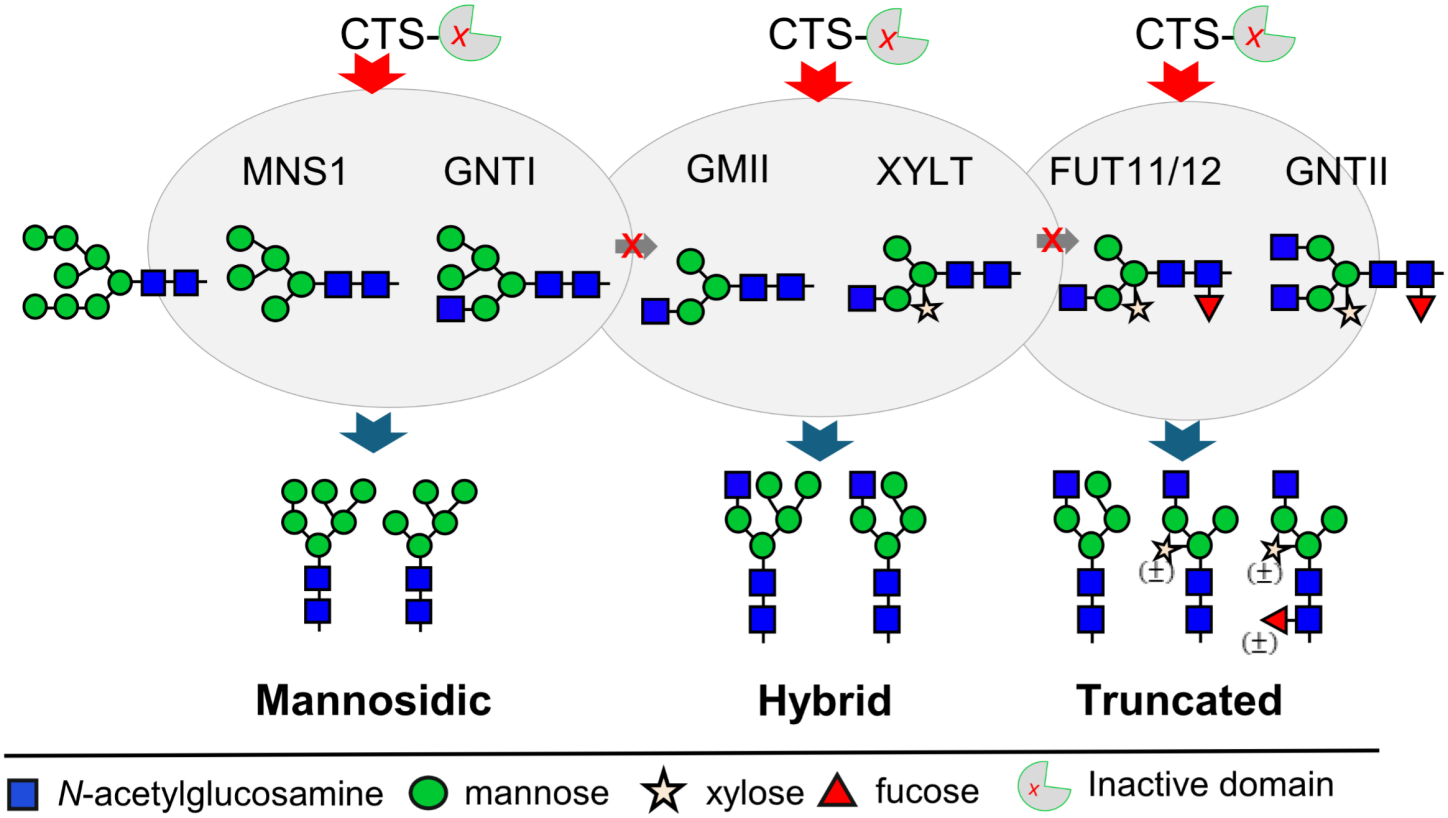
Potential strategy to interfere with N-glycan maturation in plants. Overexpression of Golgi-targeted membrane anchors (CTS regions) lacking a functional catalytic domain (e.g. expressed as fluorescent fusion proteins or with an inactive catalytic domain) can disrupt endogenous glycosylation by forming non-productive complexes with native enzymes through their stem regions. This strategy, previously demonstrated using MNS1 and GNTI CTS domains to generate mannosidic glycans (Man5), could be extended to selectively block processing in the medial or medial/trans-Golgi, enabling the accumulation of hybrid or monoantennary N-glycans (Man5Gn, Man4Gn and MGn). (+/−): presence or absence of core epitopes, depending on the genetic background of the host (wild type or ΔXF lines). N-glycan structures were labelled according to the ProGlycAn nomenclature (Proglycan_nomenclature_2023.pdf).

To promote the synthesis of high-mannose-type N-glycans (Man7-9), which resemble those found on many helminth glycoproteins and are desirable for the production of recombinant vaccine antigens in plants, strategies such as ER retention of the glycoprotein or treatment with kifunensine, have been successfully employed (Roychowdhury et al., 2018; Izadi et al., 2023a). Similarly, inhibition of GMII using swainsonine has been used to generate Man5 glycans (Choi et al., 2018), which can also be produced by expressing glycoproteins in GNTI-deficient plants, as previously discussed. However, precise engineering of plant systems to produce defined mannosidic or hybrid glycans remains challenging, as current glycoengineering strategies still require further optimization. One possible strategy to control N-glycan processing could be the use of chimeric proteins carrying specific CTS regions that interfere with glycan maturation at a defined step. Mislocalization or interaction of exogenous glycosyltransferases with endogenous enzymes via their stem regions can modulate N-glycan processing by limiting downstream enzyme access. For instance, GNTI, a key enzyme in the cis/medial-Golgi, initiates hybrid N-glycan formation by transferring a GlcNAc residue to Man5 (**Figure 1**). This terminal GlcNAc is essential for subsequent processing by medial- and trans-Golgi glycosyltransferases to form complex N-glycans. Interestingly, overexpression of the CTS region from GNTI and MNS1 fused to mRFP was shown to disrupt normal glycan processing, resulting in the accumulation of Man_5_ structures on recombinant glycoproteins that would otherwise carry complex glycans (GnGnXF) (Schoberer et al., 2023). This pioneering work demonstrated that transient expression of specific CTS domains can redirect glycosylation from complex to mannosidic forms. A similar approach could be employed to selectively block further processing in the medial-Golgi, facilitating the production of hybrid or mono-antennary N-glycans by preventing recognition by downstream glycosyltransferases (**Figure 4**). Supporting this concept, targeting of B4GALT1 to the medial-Golgi (^XYLT^B4GALT1 and ^FUT11^B4GALT1) resulted in the formation of hybrid (Man_4_A and Man_5_A) and mono-antennary (MA) β1,4-galactosylated N-glycans (**Supplementary Table S1**) (Dicker et al., 2015). Thus, the overexpression of specific CTS regions fused to an inactive catalytic domain could promote the synthesis of unprocessed glycans (M5Gn, Man4Gn and MGn, **Figure 4**).

### 4.3 Galactosylation and galactosidases

In plants, the only galactosylated native N-glycan structure is the Lewis A (Le^a^) motif. These galactosylated glycans result from the activity of GALT1 that transfers galactose in β1,3-linkage to terminal GlcNAc residues, followed by the action of α1,4-fucosyltransferase (FUT13), which adds fucose in an α1,4-linkage to GlcNAc, thereby completing the synthesis of the Le^a^ epitope (Strasser et al., 2007a) (**Figure 1**, **Supplementary Table S1**). Le^a^‐containing N‐glycans are present only on a limited number of, mostly unidentified, glycoproteins in plants, and their biological significance remains unclear (Wilson, 2001; Beihammer et al., 2021). Although rare on endogenous plant proteins, Le^a^ structures are frequently found on complex N-glycans of some plant-produced recombinant glycoproteins, such as human erythropoietin (EPO), SARS-CoV-2 spike protein, and hepatitis B virus (HBV) antigen, even when absent on the native versions of these proteins (Weise et al., 2007; Parsons et al., 2012; Jez et al., 2013; Balieu et al., 2022; Pantazica et al., 2023). Notably, overexpression of GALT1 significantly increases the abundance of Le^a^-containing N-glycans on a monoclonal antibody, without the need to overexpress FUT13 (Nguyen et al., 2023).

Le^a^ determinants are sometimes referred to as human-type glycosylation, since both Le^a^ and sialyl-Le^a^ epitopes are significantly elevated in the sera of patients with colon cancer (Terracciano et al., 2005). However, they are rarely present on glycoproteins in healthy adult humans. To eliminate Le^a^ formation in plants, the synthesis pathway has been successfully disrupted in *Physcomitrella patens* by knocking out the α1,4-fucosyltransferase and β1,3-galactosyltransferase genes (Parsons et al., 2012).

In contrast to plant β1,3-galactosylated N-glycans, mammalian and helminth glycoproteins carry N-glycans extended with antennary β1,4-galactose residues. This widespread glycan modification can significantly influence the efficacy of therapeutic glycoproteins. For instance, Fc galactosylation has been shown to affect the conformation of IgG1 and its binding to Fc receptors (Houde et al., 2010; Raju and Jordan, 2012). Moreover, β1,4-galactosylated N-glycans serve as essential acceptor substrates for terminal sialylation, a modification known to enhance glycoprotein stability, prolong serum half-life, and improve therapeutic performance (Chia et al., 2023). However, the efficient synthesis of bi-antennary β1,4-galactosylated N-glycans (AA) varies depending on the target protein (Kriechbaum et al., 2020). In fact, except for monoclonal antibodies, achieving homogeneous human-like β1,4-galactosylation in recombinant glycoproteins remains challenging. Efforts to fine tuning the expression of B4GALT1 included CTS swapping to target the enzyme to the late Golgi (**Table 3**) and optimizing expression levels through selection of weaker promoters, as discussed above. These approaches have enabled improved synthesis of sialylated N-glycans as well as Lewis X (Le^X^) and blood group structures on plant-produced glycoproteins.

The synthesis of Le^X^ ([AF], **Supplementary Table S1**) structures found on native helminth glycoproteins was achieved through co-expression of chimeric ^ST6^B4GALT1 and ^ST6^FUT9a (**Figure 2A**) (Wilbers et al., 2017). Additionally, two fucosyltransferases from the parasite *Schistosoma mansoni*, FucTD and FucTE, have also been shown to synthesize Le^X^ (van Noort et al., 2020). The predominance of monoantennary Le^X^-carrying N-glycans suggests that overexpression of GNTII may be necessary to enhance glycan branching and support bi-antennary Le^X^ formation, as discussed above (Wilbers et al., 2017).

Apart from the common β1,4GalGlcNAc (LacNAc or LN) motif, many invertebrates, such as helminths, express glycans containing GalNAc, an amino sugar derivative of galactose, in the form of LDN motifs (**Supplementary Table S1**), which also occur in vertebrates and are present on several mammalian hormones.

Engineering GalNAc-carrying glycans in plants was first explored to produce mammalian mucin-type O-glycans (Daskalova et al., 2010; Yang et al., 2012; Castilho et al., 2012) by introducing the enzymatic machinery to transfer GalNAc residues to O-glycosylation sites of recombinant proteins. These studies revealed that, among the three proteins involved in the process (UDP-GlcNAc C4 epimerase, Golgi UDP-GalNAc transporter, and β1,4-N-acetylgalactosaminyltransferase), only GalNAcT was strictly necessary for GalNAc-glycan synthesis (Castilho et al., 2012; Dicker et al., 2016). This was later confirmed by the successful synthesis of LDN on the N-glycans of *S. mansoni* kappa-5 in *N. benthamiana* using only a *C. elegans* GalNAcT (CeGalNAcT) (Wilbers et al., 2017) (**Figure 2A**). This shows that, even though GalNAc is not native to plant N-glycans, UDP-GalNAc is present within the correct Golgi compartment and can be incorporated into the antennae of N-glycans of recombinant glycoproteins. Plant-derived LDN motifs can be further modified by adding an α1,3-fucose to the distal GlcNAc (via FucT9a, SmFucTD, or SmFucTE) to yield LDN-F and an additional α1,3-fucose to the terminal GalNAc (via SmFucTF) to produce F-LDN-F, resulting in mono- and bi-fucosylated LDN motifs (**Figure 2A**, **Supplementary Table S1**) (Wilbers et al., 2017; van Noort et al., 2020; van der Kaaij et al., 2022; Zwanenburg et al., 2023). Fucosylation of LDN motifs has been shown to enhance the accumulation of GalNAc-containing N-glycans, likely by increasing resistance to HEXO-mediated removal of unsubstituted GlcNAc and/or GalNAc residues (Strasser et al., 2007a).

The ABH(O) blood group antigens are specific glycan determinants on glycoproteins and glycolipids that also carry GalNAc residues, which are crucial for their antigenicity and the determination of blood types (Stowell and Stowell, 2019). In plants, synthesis of blood group A (type 1 and type 2) structures was recently achieved on the RBD of the SARS-CoV-2 spike protein expressed in *N. benthamiana* (Konig-Beihammer et al., 2021). These modifications were generated by co-expressing either the native *A. thaliana* GALT1 (type 1) or a chimeric ^ST6^B4GALT1 (type 2) with a human α1,2-fucosyltransferase targeted to late Golgi (^ST6^FUT1 or ^ST6^FUT2) to produce H antigens (α1,2FucGalβ-R), followed by extension with α1,3-GalNAc residues via N-acetylgalactosaminyltransferase (ABO A). Interestingly, in this case co-expression of a *Yersinia enterocolitica* UDP-GlcNAc 4-epimerase (YeGNE), which converts UDP-GlcNAc to UDP-GalNAc, and a *Caenorhabditis elegans* UDP-GlcNAc/UDP-GalNAc transporter (Castilho et al., 2012) further improved the biosynthesis of blood group A type 2 trisaccharide structures (Beihammer et al., 2021) (**Figure 2A**, **Supplementary Table S1**). The efficiency of ABH antigen synthesis appears to depend on glycosite accessibility. Mono-antennary structures suggests partial removal of terminal galactose and GalNAc by endogenous apoplastic galactosidases (Kriechbaum et al., 2020), whereas terminal fucosylation protects these residues from enzymatic trimming, as also observed for fucosylated LDN motifs (Wilbers et al., 2017). Despite significant efforts to optimize terminal galactosylation, many plant-derived glycoproteins still display incompletely processed N-glycans with heterogeneous β1,4-galactosylation due to trimming of terminal galactose residues in the apoplast (Castilho et al., 2014; Wilbers et al., 2017; Bohlender et al., 2020; Kriechbaum et al., 2020). Consequently, studies assessing the impact of glycosylation on protein function or activity (other than monoclonal antibodies) rarely include β1,4-galactosylated variants (Jez et al., 2013; Schneider et al., 2014; Loos et al., 2014; Montero-Morales et al., 2019).

β-galactosidases (BGALs) exhibit specificity for β1,3-, β1,6-, or β1,4-galactosidic linkages (Ahn et al., 2007). In *N. benthamiana*, BGAL1 (NbBGAL1) is an active enzyme targeting both N- and O-glycans, capable of removing β1,4-linked galactose from N-glycans as well as terminal β1,3-galactose from Lewis-A epitopes and T-antigen O-glycans (Kriechbaum et al., 2020).

Recombinant proteins expressed in *N. benthamiana* exhibit varying sensitivity to β-galactosidases activity. While glycans on the IgG Fab fragment are fully exposed to the surrounding solvent, those in the Fc fragment are largely shielded by the opposing CH2 domain, making galactosylation at this site inherently more efficient. Interestingly, similar to HEXO3 sensitivity, the susceptibility of Fc glycans to β-galactosidase activity appears to be influenced by plant-specific core α1,3-fucosylation (Kriechbaum et al., 2020). This plant-specific modification seems to relax N-glycan or protein structural constraints, rendering terminal sugar residues more accessible to glycosidases, a phenomenon once again not reproduced by core α1,6-fucosylation (Shin et al., 2017; Kriechbaum et al., 2020).

Transient downregulation via RNA interference (RNAi) or complete knockout of *NbBGAL1* significantly reduces β-galactosidase activity, leading to higher levels of fully bi-galactosylated complex N-glycans on multiple plant-produced glycoproteins (Kriechbaum et al., 2020). More recently, NbBGAL3B was identified as a second major contributor to undesired β-galactosidase activity, displaying similar specificity for β1,4-linked galactose on N-glycans (van der Kaaij et al., 2025). BGALs hydrolyze terminal β-D-galactosyl residues from diverse substrates, including polysaccharides and glycoproteins, playing key roles in cell wall structure and function (Sampedro et al., 2012; Hoang et al., 2023). Consequently, knockout of *β*-galactosidase genes can markedly affect plant development due to their essential biological functions. In transient glycoengineering, approaches to achieve near-complete galactosylation on glycoproteins prone to galactosidase trimming have focused on exploiting the protective effect of capping sugars. This was demonstrated by producing recombinant proteins carrying terminal fucose or sialic acid residues, followed by selective removal of the capping sugars with specific fucosidases or sialidases to expose terminal β1,4-galactose (Castilho et al., 2014; van der Kaaij et al., 2025).

*De novo* β1,4-galactosylation of plant N-glycans provides a substrate for further elongation (e.g., blood group antigens and sialylation), but these newly introduced β1,4-linked galactoses can also serve as anchors for other sugars, leading to the formation of aberrant N-glycans. Several studies have shown that recombinant proteins co-expressed with B4GALT1 carry β1,4-galactosylated N-glycans decorated with additional pentoses and hexoses (Bohlender et al., 2020; Kittur et al., 2020). In *N. tabacum* and *Physcomitrella patens*, multiple pentosylations on β1,4-galactosylated N-glycans were identified as α-linked arabinoses (Bohlender et al., 2022). This modification likely arises because the β1,4-linked galactose creates a non-native acceptor site for endogenous arabinosyltransferases, which normally target arabinose to cell wall polysaccharides but can act promiscuously on engineered N-glycans. Arabinoses are absent in humans and therefore potentially immunogenic (Leonard et al., 2005). In addition, they may interfere with the full N-glycan humanization of plant-derived glycoproteins. Comprehensive characterization of this aberrant N-glycosylation and identification of the responsible glycosyltransferases remain essential future steps to enable their elimination and ensure the safety of biopharmaceuticals produced in plant-based systems.

### 4.4 Branched and bisected N-glycans

An essential step for generating complex human-type glycans is the formation of multi-antennary structures, common in human but absent in plant N-glycans. As stated above, transgenic Arabidopsis and *N. benthamiana* plants expressing the human GNTIV or/and GNTV enable the synthesis of proteins with tri- and tetra-antennary N-glycans (Gn[GnGn], [GnGn]Gn and [GnGn][GnGn]) (**Figure 2A**, **Supplementary Table S1**), when the enzymes are targeted to the medial-Golgi via CTS swapping (^FUT11^GNTIV, ^FUT11^GNTV and ^XYLT^GNTIV, ^XYLT^GNTV, **Table 3**) (Nagels et al., 2011; Nagels et al., 2012). Low abundance of multi-antennary structures on endogenous proteins may reflect HEXO activity toward β1,2-, β1,4-, and β1,6-linked GlcNAc residues (Nagels et al., 2012). Transient expression of GNTIV and V confirmed that medial-Golgi targeting resulted in more homogeneous multi-antennary glycans on recombinant proteins such as Butyrylcholinesterase (BChE), A1AT, and EPO, though efficiency depended on protein and glycosylation site accessibility (Castilho et al., 2011b; Schneider et al., 2014; Castilho et al., 2014). For example, a monoclonal antibody with an additional glycosylation site in the Fab region (such as Cetuximab), and an EPO protein fused to the Fc domain of an IgG1 (EPO-Fc), showed differing levels of accessibility to ^FUT11^GNTIV, ^XYLT^GNTIV and ^FUT11^GNTV. While glycans on the Fab region and on EPO were successfully branched with GlcNAc residues, the N-glycosylation site on the Fc fragment remained unmodified (Castilho et al., 2011b, Castilho et al., 2015).

Addition of a bisecting GlcNAc to the β-mannose of the tri-mannosyl core via a β1,4-linkage, catalyzed by GNTIII, represents another critical branching step (**Figure 2A**). Unlike other branches, bisecting GlcNAc is not elongated and often inhibits further processing by GnTIV/V and other glyco-processing enzymes (Schachter, 1986). This was demonstrated in early efforts to produce bisected N-glycans by expressing full-length human GNTIII in *Nicotiana tabacum* and BY2 cells, which primarily resulted in the formation of hybrid bisected glycans (Cox et al., 2006; Rouwendal et al., 2007; Karg et al., 2010). Similarly, the expression of chimeric GNTIII targeted to the medial Golgi (^GMII^GNTIII, ^XYLT^GNTIII and ^FUT11^GNTIII) in wild-type plants led to the production of incompletely processed N-glycans lacking core fucose and xylose (Man5Gnbi, **Supplementary Table S1**) (Castilho et al., 2011a; Castilho et al., 2011b). In contrast, fully processed bisected N-glycans were achieved when GNTIII was targeted to the trans-Golgi (^ST^GNTIII, **Table 3**) (Castilho et al., 2011a; Castilho et al., 2011b; Castilho et al., 2015). Finally, co-expression of the sequentially targeted ^XYLT^GNTV, ^FUT11^GNTIV, and ^ST^GNTIII enabled the synthesis of branched and bisected glycans on recombinant proteins with accessible glycosylation sites, such as human transferrin and EPO (Castilho et al., 2011b). As observed with other glycoengineering strategies, accessibility of Fc glycans to branching and bisecting enzymes significantly improved with α1,3-core fucosylation (FUT11), whereas α1,6-core fucosylation (FUT8) had a weaker effect (Castilho et al., 2011a; Castilho et al., 2015).

### 4.5 Bi-, multi- and poly-sialylated N-glycans

Sialylation, the attachment of sialic acid residues to glycoproteins, is a critical modification for therapeutic proteins, enhancing their stability, half-life, and biological activity (Zhu et al., 2024). It plays a vital role in various cellular processes and interactions, making it a key focus in the development and optimization of biopharmaceuticals. Ensuring proper sialylation can significantly improve the therapeutic efficacy and pharmacokinetics of glycoprotein drugs (Jeong et al., 2008; Bork et al., 2009). The broader applications of therapeutic N-glycan engineering are driven by the goal of optimizing α2,3- and α2,6-linked sialylation of glycoproteins (Zhong et al., 2022). Among these efforts, α2,6-sialylation has garnered particular attention in glycoengineering, especially in the context of antibodies. Research indicates that the anti-inflammatory activity of antibodies is largely attributed to Fc-sialylation, highlighting the significance of this modification in enhancing therapeutic efficacy (Anthony et al., 2008).

*De novo* sialic acid biosynthesis occurs in the cytosol through a four-step pathway. The key enzyme UDP-GlcNAc 2-epimerase/ManNAc kinase (GNE) initiates the process by converting UDP-GlcNAc into ManNAc-6P, which is subsequently condensed with phosphoenolpyruvate (PEP) by Neu5Ac-9-phosphate synthase (NANS) to form Neu5Ac-9P. This intermediate is then dephosphorylated by Neu5Ac-9-phosphate phosphatase (NANP) to yield free N-acetylneuraminic acid (Neu5Ac). Neu5Ac is activated in the nucleus by CMP-Neu5Ac synthetase (CMAS) to form CMP-Neu5Ac, which is then transported into the Golgi lumen via the CMP–sialic acid transporter (CSAT). In the final step, α2,3- or α2,6-sialyltransferases (ST3GAL4 and ST6GAL1) transfer Neu5Ac from CMP-Neu5Ac to β1,4-galactosylated acceptor substrates in the trans-Golgi, thereby completing the synthesis of complex-type mammalian N-glycans (Zhu et al., 2024).

Since plants naturally lack this pathway and its corresponding enzymes, engineering sialylation requires extensive metabolic and subcellular modifications to enable the formation of human-type sialylated glycans. Fine-tuning of the plant sialylation pathway commonly includes the use of a truncated CMAS variant lacking the 40 N-terminal amino acids, which destabilizes the enzyme, and a feedback-insensitive GNE mutant (GNE^R263L^) to circumvent CMP-Neu5Ac–mediated negative feedback regulation (Castilho et al., 2010; Kallolimath et al., 2016; Bohlender et al., 2020). Interestingly, stable or transient co-expression of GNE, NANS, and CMAS in plants results in the accumulation of Neu5Ac rather than Neu5Ac-9-P, indicating that an endogenous plant phosphatase with NANP-like activity catalyzes the dephosphorylation step (Castilho et al., 2008; Castilho et al., 2010). However, co-expression of NANP can further increase Neu5Ac production (Bohlender et al., 2020).

In the past years several approaches have been performed to establish sialylation in plants. In *N. benthamiana* functional *N-*glycan sialylation could be achieved by MUTE of the sialylation pathway-genes (Izadi et al., 2023a). This resulted in an efficient sialylation (up to 90%) of several recombinant proteins produced in plants (Castilho et al., 2010; Castilho et al., 2014; Jez et al., 2013; Schneider et al., 2014; Kallolimath et al., 2016; Goritzer et al., 2019; Montero-Morales et al., 2019; Kallolimath et al., 2020; Izadi et al., 2025).

For proteins with exposed glycosylation sites, terminal sialic acid protects against trimming by BGAL1 and HEXO3. Accordingly, full galactosylation on these sites can be achieved by sialidase treatment (Kriechbaum et al., 2020). In contrast, the impact of core α1,3-fucosylation on sialylation of glycosylation sites with difference exposure has been demonstrated with cetuximab, which carries N-glycans in both the Fab and CH2 domains. While glycan processing in the Fab domain is unaffected by fucosylation, the presence of plant-specific α1,3-core fucose markedly increases the levels of bi-antennary sialylated Fc glycans (Castilho et al., 2015; Kallolimath et al., 2016; Kallolimath et al., 2020; Eidenberger et al., 2022), an effect not reproduced by the over-expression of mammalian α1,6-core fucosylation (FUT8) where mainly monosialylated N-glycans are detected (ANaF, **Supplementary Table S1**) (Castilho et al., 2015).

Among the genes required to establish sialylation in plants, the correct subcellular localization of B4GALT1 and of ST3GAL4 or ST6GAL1 represents a major bottleneck. In moss, co-expression of B4GALT1 and ST6GAL1 targeted to the late Golgi by CTS swapping with FUT13 (FT4) led to the synthesis of mono-antennary sialylated glycans (MNa, **Supplementary Table S1**) (Bohlender et al., 2020).

Specific glycosylation patterns, including the degree of branching and sialylation, can modulate the biological activity of glycoproteins and enhance their pharmacokinetic properties (Teare et al., 2019). Overexpression of GNTIV and GNTV, increases the glycan antennary structure and potentially the number of terminal GlcNAc residues available for sialylation. For example, the introduction of tri-antennary sialylated glycans on EPO significantly enhanced its hematopoietic activity *in vivo* (Yin et al., 2015). In plants, the combined expression of GNTIV and GNTV with genes required for protein α2,6-sialylation enabled the production of tri- and tetra-antennary sialylated N-glycans on several recombinant proteins (**Figure 2A**, **Table 3**) (Castilho et al., 2012; Castilho et al., 2013; Castilho et al., 2014; Schneider et al., 2014).

Glycans are typically mono-sialylated, but sialic acids can also form homo-polymers of α2,8- or α2,9-linked residues known as polysialic acid (PSA) (Colley et al., 2014). Among the few naturally polysialylated proteins identified, the neural cell adhesion molecule (NCAM) is the best studied, carrying α2,8-linked NeuAc PSA on two of its N-glycans (Close et al., 2003). The addition of PSA to proteins, termed polysialylation (polySia), is a complex post-translational modification that can improve the pharmacokinetic behavior of therapeutic proteins while reducing their immunogenicity. Unlike synthetic polymers such as PEG, PSA is biodegradable and non-immunogenic, making it a safer alternative for therapeutic protein modification (Zhang et al., 2023; Chia et al., 2023).

Recent advances in biosynthetic engineering have enabled the polysialylation of recombinant proteins in both bacteria (Keys et al., 2017) and plants (Kallolimath et al., 2016). In bacterial systems, a biosynthetic pathway for site-specific polysialylation was established in *Escherichia coli*, in which a cytoplasmic polypeptide-glycosyltransferase installs a defined primer onto the target protein, followed by elongation with bacterial glycosyltransferases to generate long-chain polySia. This strategy has been successfully applied to modify green fluorescent proteins and therapeutic DARPins (designed ankyrin repeat proteins) (Keys et al., 2017).

In *N. benthamiana*, transient co-expression of two human polysialyltransferases (polySTs: ST8SiaII and ST8SiaIV) together with genes required for α2,6-sialylation demonstrated that the synthesis of biologically active α2,8-linked PSA is feasible (**Figure 2A**) (Kallolimath et al., 2016). Bacterial polysialyltransferases are generally more promiscuous than their mammalian counterparts (Willis et al., 2008; Lindhout et al., 2011), and engineered bacterial variants capable of generating oligo- and polysialic acids of defined length represent promising candidates to further enhance in planta synthesis of site-specific polySia on diverse recombinant glycoproteins.

Although substantial progress has been made in plants and other heterologous systems, several biochemical and engineering challenges remain. These include achieving precise control over polymer length, ensuring efficient transfer of defined polySia to target proteins, and optimizing downstream purification processes to handle highly hydrophilic polysialylated products. Continued development in biosynthetic pathway engineering, enzyme design, and analytical methods will be critical to fully realize the potential of polysialylation in plant-based expression platforms.

## 5 Final remarks

Advancements in glycoscience and the development of a comprehensive network in glycosylation pathways are creating new opportunities for designing next-generation therapeutic proteins with tailored glycosylation profiles (Zhong et al., 2022).

Glycans are intrinsically flexible molecules that can adopt many conformations (Yadav et al., 2025). In complex N-glycans, the core Man_3_GlcNAc_2_ structure has two arms: the α1,3-arm linked to the mannose at the 3-position and the α1,6-arm linked to the mannose at the 6-position. Molecular dynamics simulations have revealed that the α1,3-arm tends to be more extended and accessible to glycosyltransferases in the Golgi apparatus, whereas the α1,6-arm often folds closer to the protein surface or the core, reducing its accessibility (Andre et al., 2007; Andre et al., 2009). Kinetic factors further influence this pattern, as modification of the α1,3-arm can sterically hinder subsequent modification of the α1,6-arm. These structural differences are crucial for the efficiency of glycosylation processes.

The addition of specific sugars to the core glycan structure (e.g., sialic acids, fucose, GlcNAc) can induce conformational shifts that alter the glycan’s shape and exposure. For instance, addition of sialic acid in α2,6-linkage to galactose can cause the glycan arm to bend outward, increasing accessibility to lectins (Stadlmann et al., 2010). Also, core fucosylation and the introduction of bisecting GlcNAc residues have been shown to induce long-range effects on glycan conformation (Nishima et al., 2012; Thaysen-Andersen and Packer, 2012). Specifically, core fucosylation significantly alters the conformational equilibria and strongly influences glycan structure and flexibility. Structural and molecular dynamics studies show that these effects depend more on glycan accessibility than on the peptide backbone (Andre et al., 2009; Nishima et al., 2012) and that glycan modifications occur in a branch-specific manner (Barb et al., 2009). Crystal structures of human IgG1 Fc glycoforms further revealed that core fucosylation pushes glycan arms outward, altering their positioning and accessibility (Ferrara et al., 2011).

**Figure 5:**
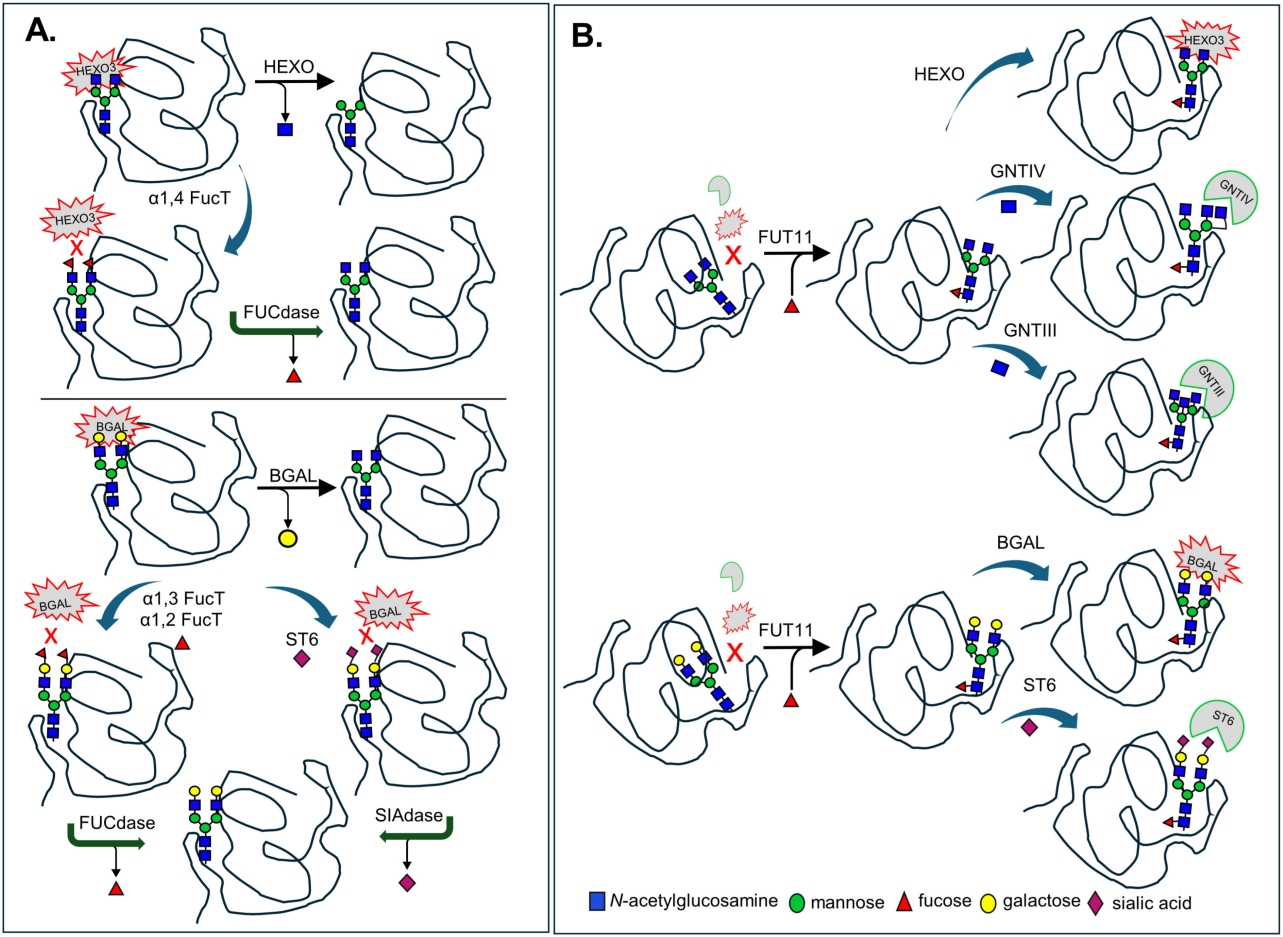
Role of fucosylation in modulating accessibility and processing of exposed and buried N-glycosylation sites. **(A)** Exposed glycosylation sites are susceptible to hydrolytic trimming by endogenous glycosidases such as HEXO and BGAL1. Capping terminal sugars with terminal fucose residues (or with sialic acid in the case of terminal galactose) protects them from enzymatic removal. This strategy, combined with subsequent *in vitro* removal of fucose (or sialic acid) using specific glycosidases, enables the accumulation of fully processed complex N-glycans (e.g., GnGn or AA structures). **(B)** In contrast, buried glycosylation sites (such as those found in Fc domains of antibodies) are shielded from hydrolytic enzymes but may also be inaccessible to certain glycosyltransferases. In such cases, core fucosylation, particularly plant-specific α1,3-fucose, appears to induce a rotation of the chitobiose core that renders the α1,6-arm branch accessibility to glyco-processing enzymes (both glycosidases and glycosyltransferases). This approach has proven effective for modulating antibody glycosylation toward bisected, branched, and fully sialylated human-like structures. Combining this strategy with subsequent *in vitro* removal of core fucose can yield fully humanized glycans devoid of plant-specific epitopes.

More recently, structural insights into complex human and plant N-glycans demonstrated that plant-specific α1,3-fucose induces a rotation of the chitobiose core (β1,4GlcNAc-GlcNAc), thereby promoting an open conformation of the α1,6-arm branch. In contrast, in α1,6-core fucosylated N-glycans, the α1,6-arm is predominantly in a folded conformation (Fogarty et al., 2020). These conformational effects suggest that plant-specific core α1,3-fucosylation can relieve N-glycans from structural constraints (e.g. Fc glycans), increasing the accessibility of terminal sugars to both glycosyltransferases (e.g., GNTs and STs) and glycosidases (e.g., HEXO3, BGALs), an effect not mimicked by human-like core α1,6-fucosylation (Castilho et al., 2015). Additionally, capping exposed N-glycans with α1,2-, α1,3- and α1,4-linked fucose can protect against trimming by glycosidases, which can be exploited to create tailored glycoforms via *in planta* fucosylation combined with *in vitro* enzymatic treatment (Leonard et al., 2009) (**Figure 5**).

Extending this strategy to remove core α1,3-fucose residues to achieve complete humanization of plant-derived N-glycans remains challenging, as core-specific α1,3-fucosidases have not yet been identified. Although a GH29 α-fucosidase from *Omnitrophica* bacterium, which shows a strong preference for hydrolyzing α1,6-linked core fucose, can also remove the core α1,3-fucose residue from plant complex-type N-glycans (Vainauskas et al., 2018), its biotechnological application for selective core fucose removal warrants further investigation.

Decorating plant-produced glycoproteins with non-human or pathogen-mimicking glycans provides a strategic means to enhance vaccine immunogenicity. For instance, incorporation of plant-specific β1,2-xylose and α1,3-fucose, as well as Lewis-A or helminth-like motifs such as LacdiNAc and fucosylated LDN, can act as self-adjuvants, boosting both humoral and cellular immune responses. This strategy allows the simultaneous optimization of antigenicity and immunostimulatory potential, making tailored glycosylation a powerful tool in recombinant vaccine design (Rosales-Mendoza et al., 2016; Zwanenburg et al., 2023; Vo and Trinh, 2025). Therefore, while the removal of plant-specific glyco-epitopes is essential for producing human-compatible biopharmaceuticals, it is important to note that such glycans (including core α1,3-fucose) are not inherently undesirable and can be leveraged as a glycoengineering tool.

The success of plant molecular farming, particularly using *N. benthamiana* as a production platform, greatly relies on its high tolerance for genetic manipulation and glycoengineering. However, major challenges remain, including the need to prevent unintended developmental phenotypes arising from the stable “humanization” of plant glycosylation pathways. Consequently, most major advances in producing helminth- and human-like glycans in plants have been achieved via transient expression of glycan-modifying genes, enabling controlled glycoengineering without long-term effects on plant development.

While plant glycoengineering still faces several limitations, ongoing research and innovative strategies that combine *in planta* and *in vitro* approaches are steadily overcoming these challenges, including leveraging plant-specific α1,3-fucose to design glycoproteins with optimized structure and immune-modulating properties.

## 6 Outstanding challenges and future directions in plant glycoengineering

Despite major advances, several challenges still limit the precision, predictability, and scalability of plant N-glycoengineering. One persistent issue is the batch-to-batch variability observed in transient expression systems, which raises concerns for regulatory compliance and good manufacturing practices. Variability can arise from fluctuating plant growth conditions, differences in agroinfiltration efficiency, and the performance of expression constructs (Akher et al., 2025). A design-of-experiments (DoE) approach has been proposed to systematically identify and mitigate these sources of variability by optimizing environmental parameters, infiltration conditions, and expression timing (Buyel and Fischer, 2014; Buyel, 2023). Establishing more standardized and tightly controlled transient expression workflows will be essential to reduce glycan heterogeneity and improve reproducibility (Akher et al., 2025; Annese et al., 2025).

Another major challenge lies in accurately predicting glycosite accessibility and the resulting glycan maturation. Glycosylation outcomes are strongly influenced by protein three-dimensional structure, domain flexibility, and steric constraints around individual glycosylation sites. Although computational tools, including molecular dynamics simulations, provide valuable insights, current models often lack the accuracy needed to reliably forecast *in vivo* glycan processing due to high parametric complexity and the intrinsic flexibility of glycans. Improving these predictive tools will require better integration of experimental structural data, refined algorithms that capture glycan dynamics, and the incorporation of machine learning approaches.

At the cellular level, mislocalization or suboptimal localization of native and heterologous glycosyltransferases in the Golgi apparatus remain a significant bottleneck. Even with engineered targeting motifs, differences in trafficking behavior and competitive interactions with endogenous enzymes can lead to incomplete or unintended glycan processing. Developing improved strategies for controlling enzyme residency times and Golgi compartment specificity will be critical for achieving more precise pathway engineering.

Looking ahead, progress will depend on the convergence of several technological advances. Synthetic biology tools and precision genome editing can enable more robust plant expression systems, while high-throughput analytical platforms will allow rapid screening and selection of optimal glycosylation conditions. Advances in computational modeling and artificial intelligence hold promise for enabling predictive control of glycosylation pathways, bridging the gap between theoretical design and experimental outcomes. By addressing these interconnected challenges, the field of plant glycoengineering is well positioned to deliver increasingly reliable and scalable production platforms for next-generation biopharmaceuticals.
